# Whole-Genome Sequencing of Dorper × Hu Hybrid Sheep for Screening Selection Signatures Associated with Litter Size

**DOI:** 10.3390/ani15233505

**Published:** 2025-12-04

**Authors:** Liying Qiao, Ke Ma, Quanhong Yao, Siying Zhang, Zhixu Pang, Wannian Wang, Ke Cai, Wenzhong Liu

**Affiliations:** 1 Department of Animal Genetics, Breeding and Reproduction, College of Animal Science, Shanxi Agricultural University, Taigu 030801, China; liyingqiao1970@163.com (L.Q.); mk753951486250@163.com (K.M.); yqh978312@163.com (Q.Y.); zhangsy9526@163.com (S.Z.); pang_z_x@163.com (Z.P.); wannian1876@163.com (W.W.); caik1021@163.com (K.C.); 2 Key Laboratory of Farm Animal Genetic Resources Exploration and Precision Breeding of Shanxi Province, Taigu 030801, China

**Keywords:** Dorper × Hu hybrid sheep, litter size, selective sweep analysis, whole-genome resequencing

## Abstract

Sheep are an economically important livestock species, and reproductive performance is a key trait affecting productivity. The Dorper × Hu hybrid sheep (DHS), widely bred in China, provides a valuable model for studying the genetic basis of prolificacy. This study aimed to investigate the genomic architecture and identify candidate genes associated with high litter size in DHS using whole-genome selective sweep analysis and genome-wide association study (GWAS). A total of 31 DHS individuals with complete reproductive records were sequenced and compared with publicly available genomic data from 20 Hu sheep (HUS) and 10 Dorper sheep (DPS). Population genetic structure and diversity were assessed using phylogenetic trees, principal component analysis (PCA), and ADMIXTURE analysis. To identify key genomic regions associated with litter size, we performed selective sweep analysis between the polytocous and monotocous subpopulations of DHS using multiple methods within a 50 kb sliding window framework, including $F_{ST}$, $\theta_{\pi }$ ratio, XP-CLR, and XP-EHH; we also conducted GWAS. DHS exhibited a distinct genetic structure with admixed ancestry and elevated genetic diversity. Genetic diversity analysis showed that DHS retained moderate levels of heterozygosity and polymorphism, comparable to or exceeding those of its parental breeds. Comparative analysis between polytocous and monotocous DHS identified reproduction-associated genes, including *MUC1*, *PLCB4*, *SIN3A*, and *ELAVL2*, enriched in pathways such as ovarian steroidogenesis, insulin secretion, and circadian entrainment. Furthermore, genome-wide association study (GWAS) identified 140 significant loci (*p* < 10^−5^) associated with reproductive traits. From these, 10 candidate SNPs were selected for validation through single-marker association analysis in 200 DHS individuals, among which two loci—g.88680390 C>A (*SLC24A2*/*MLLT3*) and g.18197516 T>C (*ABCA1*)—showed significant correlations with litter size. These findings enhance our understanding of the genetic basis of prolificacy in DHS and provide valuable molecular markers for genomic selection in sheep-breeding programs.

## Simple Summary

Reproductive performance is a key factor influencing sheep production efficiency. The Dorper × Hu hybrid sheep (DHS) combines the rapid growth of Dorper sheep with the high fecundity of Hu sheep and is widely used for lamb production in China. In this study, whole-genome selective sweep analysis and genome-wide association study (GWAS) were used to explore the genetic basis of high fecundity in DHS. The results revealed that DHS possesses a distinct genetic structure and high genetic diversity derived from its parental breeds. Several candidate genes related to reproduction were identified. Moreover, two SNPs—g.88680390 C>A (*SLC24A2/MLLT3*) and g.18197516 T>C (*ABCA1*)—were significantly associated with litter size. These findings provide valuable molecular markers for improving reproductive efficiency and advancing genomic breeding in meat sheep.

## 1. Introduction

Sheep (*Ovis aries*) have served an essential source of meat, wool, and milk for thousands of years. China has a long history of sheep domestication and boasts abundant resources of indigenous breeds, which are generally categorized into three main groups: Mongolian, Kazakh, and Tibetan sheep [1]. Through long-term domestication and selective breeding, these breeds have adapted to diverse ecological regions and evolved distinct desirable traits [2], such as high reproductive performance in Small-Tailed Han and Hu sheep, superior meat quality in Tan sheep, and strong adaptability to harsh environments in Tibetan sheep. To overcome the limitations of indigenous breeds and meet the growing demand for high-quality lamb, crossbreeding local breeds with highly productive exotic breeds followed by long-term selection has become a primary genetic strategy for improving sheep production traits [3]. In China, this approach has yielded several successful composite breeds, including Luxi Black Head sheep [4] and Bamei mutton sheep [5], both of which exhibit enhanced production efficiency. Among them, the Dorper × Hu hybrid sheep (DHS), derived from crossing Dorper sheep (DPS) with Hu sheep (HUS), has emerged as one of the dominant breeds for lamb meat production in China. DHS combines the rapid growth and excellent carcass traits of Dorper sheep with the high prolificacy and non-seasonal estrus of Hu sheep [6], making it a critical genetic resource for meat sheep improvement.

In recent years, the rapid advancement of whole-genome resequencing technology, coupled with the completion of the sheep reference genome [7], has significantly accelerated research into the genetic mechanisms underlying morphological and economic traits in sheep. Whole-genome resequencing has been widely utilized to investigate sheep domestication and evolution [8], as well as to unravel the genetic basis of complex traits—such as reproductive capacity, coat color, horn type, tail type, and body size [9]. The identification of genetic variants associated with these traits deepens our understanding of sheep biology and provides a valuable foundation for genomic selection in breeding programs [10].

Among various production traits, reproductive efficiency plays a pivotal role in determining productivity and economic return in sheep production systems. It is regulated by genetic, hormone, environmental, and management factors. However, litter size is a complex trait with low to moderate heritability [11], controlled by both major genes and polygenes and influenced by environmental factors. Several major genes, such as *BMPR1B* [12], *BMP15*, and *GDF9* [13]—have been shown to significantly affect this trait. In particular, the well-characterized *FecB* mutation (*BMPR1B*: A746G/p.Q249R) markedly increases litter size, and this mutation is nearly fixed in Hu sheep [1]. The *BMP15* gene plays a vital role in follicular development by promoting granulosa cell proliferation and inhibiting follicle-stimulating hormone expression. *GDF9* is also essential for folliculogenesis, with several heterozygous mutations associated with increased litter size. Notably, candidate genes linked to prolificacy vary across breeds, reflecting the polygenic and breed-specific nature of litter size. Therefore, identifying fertility-related genes is critical for elucidating the genetic mechanisms underlying reproductive performance and laying a solid foundation for marker-assisted selection in sheep breeding.

Given that the molecular mechanisms and candidate genes regulating reproductive traits in DHS remain incompletely understood, this study integrates whole-genome resequencing data from 31 DHS individuals with publicly available genomic data from 20 HUS and 10 DPS. Through genetic diversity analysis and genetic structure analysis, we systematically clarified the phylogenetic status of DHS. Meanwhile, we compare selection signals between the polytocous subgroup (those with three consecutive multiple-lamb litters) and monotocous subgroup (those with three consecutive single-lamb litters) within the DHS population to identify candidate genes associated with high prolificacy, ultimately clarifying the key genetic variants contributing to differences in reproductive performance.

## 2. Materials and Methods

### 2.1. DNA Sample and Sequence

A total of 31 Dorper × Hu hybrid sheep (DHS), all three years of age and possessing records of three consecutive lambings, were selected from Genyuan Animal Husbandry Farm in Shanxi Province, China. This included 15 individuals that consistently delivered multiple lambs (prolific/polytocous) and 16 individuals that consistently delivered single lambs (non-prolific/monotocous) across all three parities. In Supplementary Table S1, individuals denoted with a prefix of “M” correspond to monotocous sheep (non-prolific), while those starting with “S” refer to polytocous sheep (prolific). Approximately 5 mL of jugular blood was collected from each individual into EDTA-treated tubes and stored at −20 °C until DNA extraction. To assess genetic background and admixture patterns, whole-genome resequencing data from 20 HUS and 10 DPS were obtained from the Sequence Read Archive (BioProject: PRJNA681929) for comparative analysis.

### 2.2. Whole-Genome Data Processing

Raw genome sequence data were quality filtered using fastp (v0.23.4) [14] with parameters “−n 1 −q 5 −u 50 −l 100” to remove reads containing an excessive number of ambiguous bases, low-quality reads, or adapter contamination. Clean reads were mapped and aligned to the *Ovis aries* reference genome (ARS-UI_Ramb_v2.0, https://www.ncbi.nlm.nih.gov/datasets/genome/GCF_016772045.1/, accessed on 1 May 2025) using BWA (v0.7.17) [15], and sequencing depth for each sample was calculated using SAMtools (v1.20) [16]. Variant calling was performed across all samples using the HaplotypeCaller module in GATK (v4.18) [17]. To reduce false positives, SNPs were filtered using the following thresholds: QD < 2.0, FS > 60.0, MQ < 40.0, SOR > 3.0, MQRankSum < −12.5, and ReadPosRankSum < −8.0. Additional stringent filters were applied to remove: (i) SNP clusters (more than one SNP within 5 bp), (ii) SNPs within 5 bp of indels, and (iii) genotypes with low quality (GQ < 20), which were labeled as “lowGQ”.

### 2.3. Population Structure Analysis

Population genetic structure was assessed using three complementary approaches: Neighbor-Joining (NJ) tree construction, Principal Component Analysis (PCA), and ancestry inference. NJ trees were constructed in MEGA (v11.0.13) [18] using pairwise genetic distance matrices calculated by PLINK. PCA was performed using GCTA (v1.94.1) [19] based on the genomic relationship matrix calculated using the VanRaden method. ADMIXTURE (v1.30) [20] was employed to estimate individual ancestry components, with the number of ancestral populations determined to be K = 2–3.

### 2.4. Genetic Diversity Analysis

Genetic diversity indices including observed heterozygosity (Ho), expected heterozygosity (He), polymorphic marker ratio ($P_{N}$), minor allele frequency (MAF), and polymorphism information content (PIC) were calculated using PLINK [21]. Runs of homozygosity (ROH) were identified using the following criteria: a minimum length of 300 kb, at least 50 SNPs per ROH, and a SNP density of one per 50 kb. Up to 5 missing calls and 3 heterozygous calls were allowed per window, with a maximum gap of 300 kb between adjacent SNPs. Linkage disequilibrium (LD) decay was analyzed using PopLDdecay (v3.42) [22]. The parameters used were: MaxDist 1,000,000, MAF 0.05, Het 0.9, and Miss 0.1.

### 2.5. Selective Sweep Identification

Genomic selection signals were identified using VCFtools (v0.1.16) [23] for fixation index ($F_{ST}$) and nucleotide diversity ($\theta_{\pi }$) ratios, XP-CLR (v1.1.2) [24] for cross-population composite likelihood ratio (XP-CLR), and selscan (v1.2.0) [25] for XP-EHH. All analyses were conducted using a 50 kb sliding window with a 10 kb step. Candidate regions were defined as the top 1% windows ranked by scores from each selection method. Genes located within these candidate regions were annotated and analyzed for functional enrichment using Gene Ontology (GO) and Kyoto Encyclopedia of Genes and Genomes (KEGG) pathways via DAVID [26].

### 2.6. Genome-Wide Association Study (GWAS)

A GWAS was conducted using WGS data from 31 DHS ewes (15 polytocous and 16 monotocous), all aged three years with records from three consecutive lambings. The Generalized Linear Model (GLM) implemented in GEMMA [27] was utilized to identify SNPs associated with prolificacy traits. Phenotypes were treated as a binary variable: ewes with three consecutive multiple-lamb litters were classified as polytocous (coded as 1), while those with three consecutive single-lamb litters were classified as monotocous (coded as 0). To correct for population stratification, the top ten principal components (PCs) derived from PCA were incorporated into the model.

### 2.7. Targeted Genotyping and Association Analysis

Based on production records, 200 DHS with three consecutive lambing events were selected for targeted SNP genotyping. Jugular blood samples (5 mL) were collected into EDTA-treated tubes for DNA extraction. Candidate nonsynonymous SNPs were identified. Genotyping was performed using the MassARRAY^®^ platform (Agena Bioscience, San Diego, CA, USA). DNA samples were quality-controlled prior to PCR amplification of target fragments. Single-base extension reactions were carried out using specific extension primers. The resulting products were purified, spotted onto a SpectroCHIP array, and analyzed by mass spectrometry. Genotyping was achieved by distinguishing products based on molecular weight differences. All MassARRAY genotyping assays were conducted by Beijing Compass Biotechnology Co., Ltd. Detailed primer information is provided in Table S8.

For association analysis, consistent with the GLM framework used in the GWAS (Section 2.6), SNP-trait associations were also evaluated using a GLM, but adapted for the analysis of quantitative traits (litter size) with repeated measurements. The model included parity as a fixed effect to account for its contribution to litter size variation:

$$Y=\mu +W\alpha +X_{s}\beta_{s}+e$$

where Y is the litter size, μ is the population mean, $X_{s}$ is the genotype vector for SNP s, $\beta_{s}$ is the genetic effect of SNP, α is the parity effect, W is the design matrix for α, and e is the residual error.

## 3. Results

### 3.1. Genome Resequencing and Identification of Single Nucleotide Polymorphisms

Whole-genome sequencing data were generated for 31 DHS, and publicly available genomic data from 30 sheep (10 DPS and 20 HUS; PRJNA681929 [28]) were also included in the analysis. In total, the raw sequencing data amounted to 2067.57 G, of which 2023.03 G remained after quality filtering. The average Q30 was 94.26%, and the GC content was 44.82% (Supplementary Table S1). All 61 samples were aligned to the sheep reference genome, achieving an average alignment rate of 99.86% (Supplementary Table S2) and an average sequencing depth of 10.83× (Supplementary Table S3). After variant calling and filtering, an average of 12.52 million SNPs were identified per DHS individual, 12.06 million per DPS individual, and 12.39 million per HUS individual (Supplementary Table S4). Most SNPs were located in intergenic and intronic regions, while a smaller proportion were found in exonic regions, untranslated regions (UTRs), and splicing sites. The genomic distribution density of SNPs is shown in Supplementary Figure S1.

### 3.2. Population Genetic Structure

**Figure 1:**
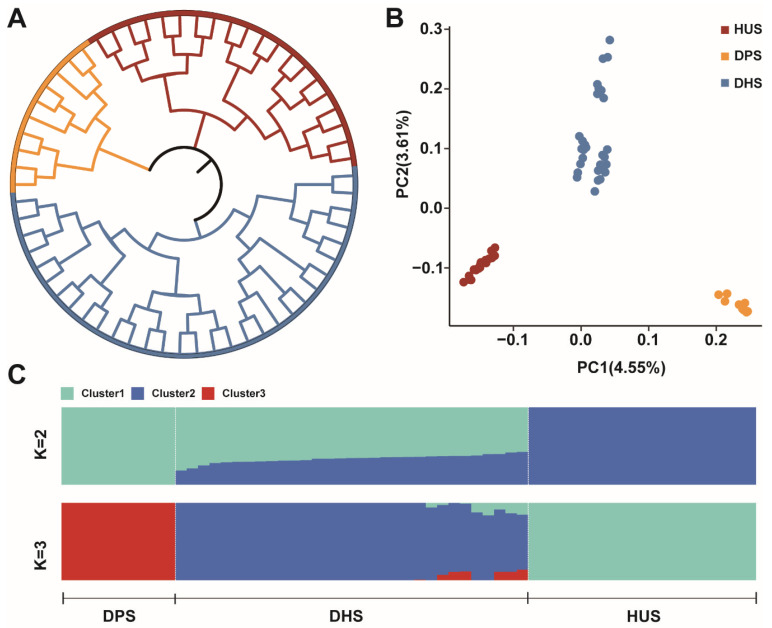
Population Genetics Analyses of Samples. (**A**) Phylogenetic trees of three sheep populations constructed based on the neighbor-joining method; (**B**) PCA results for three sheep populations; (**C**) Analysis of ancestral composition of three sheep populations.

Based on genome data, we analyzed the genetic relationships among the three sheep populations. The phylogenetic tree, constructed using individual SNP variants, revealed that DHS, DPS, and HUS were clearly grouped into three distinct genetic clusters (Figure 1A). Furthermore, PCA further supported the genetic differentiation among the three populations. PC1 and PC2 explaining 4.55% and 3.61% of the total genetic variation, respectively, and clearly separated the individuals into three clusters corresponding to DHS, DPS, and HUS (Figure 1B). Ancestry component analysis revealed that when K = 2, DPS and HUS each formed distinct ancestral groups, while DHS exhibited a higher proportion of DPS ancestry (Figure 1C). When K = 3, each population exhibited a unique genetic signature, allowing clearer differentiation among the three groups.

### 3.3. Population Genetic Diversity

**Figure 2:**
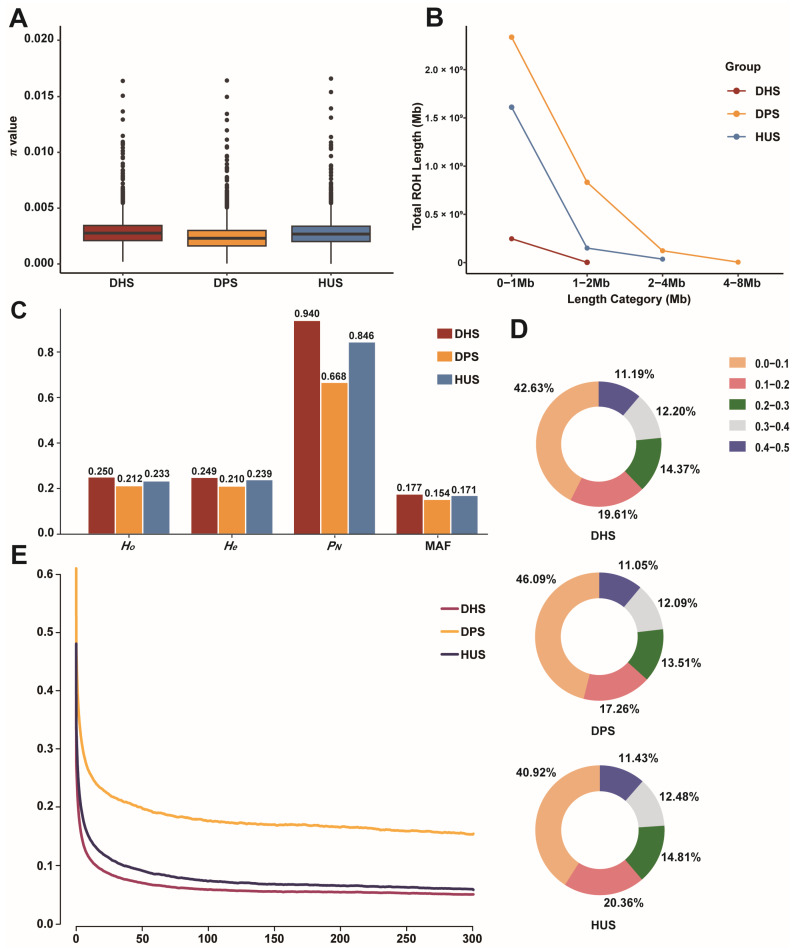
Genetic Diversity Statistics of Three Sheep Breeds. (**A**) Whole-genome distribution of π in three populations; (**B**) Distribution of total ROH lengths by length category for the three populations; (**C**) Statistics of genetic diversity parameters of the three populations, including *Ho*, *He*, *P_N_*, and MAF; (**D**) Distribution of MAF in three populations; (**E**) Genome-wide average LD decay for the three breeds.

We systematically assessed genetic diversity, polymorphism, and genome-wide patterns among the three sheep populations to investigate how hybridization has shaped the genetic background of DHS. Nucleotide diversity (π) analysis revealed that DHS exhibited diversity levels comparable to HUS and greater than DPS (Figure 2A). ROH analysis indicated that most ROHs were concentrated in the 0–1 Mb range across all populations, but DPS had significantly more ROHs in the 1–2 Mb, 2–4 Mb, and 4–8 Mb intervals (Figure 2B). The *Ho*, *He*, and MAF of DHS were 0.250, 0.249, and 0.177, respectively—values slightly higher than those observed in DPS and HUS. The *P_N_* in DHS reached 0.940, significantly higher than in DPS (0.668) and HUS (0.846), indicating that more polymorphic functional loci were retained due to its hybrid background (Figure 2C). The MAF distribution revealed that DHS had the lowest proportion of SNPs in the 0.4–0.5 interval (11.19%), a trend similar to those observed in DPS and HUS, indicating broadly comparable allele frequency patterns across the three populations (Figure 2D). LD analysis revealed that DHS exhibited lower LD levels across all genomic distances, particularly over short distances, with DPS showing the highest LD and HUS an intermediate degree (Figure 2E). These findings, consistent with the ROH patterns, suggest that hybridization in DHS increased genetic diversity, reduced linkage disequilibrium, and contributed to a broader genomic foundation conducive to future breeding improvement.

### 3.4. Selection Signatures Between the Polytocous and Monotocous Sheep

Prior to analyzing novel selection signatures for prolificacy, we first confirmed the status of the major prolificacy gene, *FecB* (*BMPR1B* c.746G>A). The prolific B allele was fixed across all 31 DHS individuals, resulting in minimal polymorphism at this locus. Specifically, the 15 polytocous individuals comprised 14 B+ and 1 BB genotypes, while the 16 monotocous individuals consisted of 13 B+ and 3 BB genotypes. Due to this high genetic fixation and insufficient polymorphism across the two groups, the *FecB* was unable to register as a significant selective sweep region in this intraspecies comparison. This necessitates the search for other novel or quantitative loci contributing to litter size variation.

**Figure 3:**
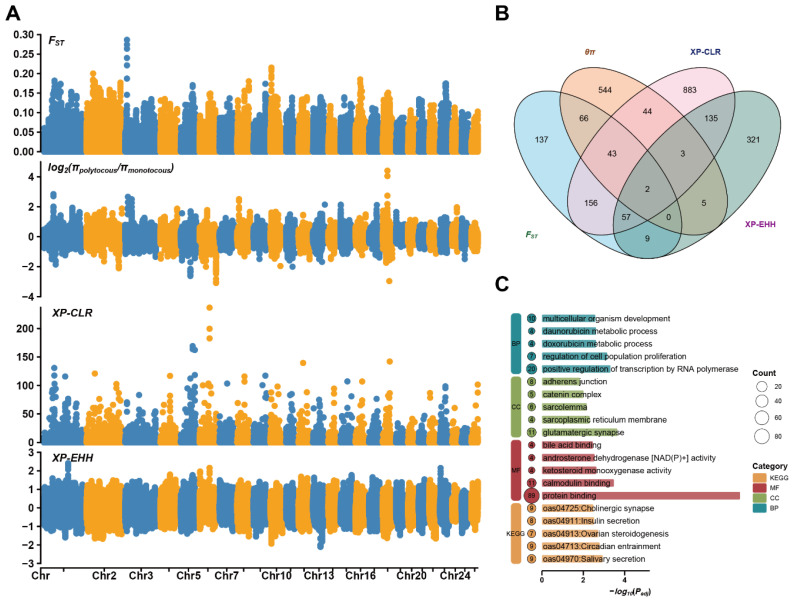
Selection signal analysis and functional enrichment of genes with litter size in DHS. (**A**) Manhattan plots of genome-wide selection signatures between polytocous and monotocous sheep, based on $F_{ST}$,$\;\log_{2}\left(\pi_{polytocous}/\pi_{monotocous}\right)$, XP-CLR, XP-EHH. Different colors indicate different chromosomes. (**B**) Venn diagram of candidate genes identified by different selection methods. (**C**) GO and KEGG enrichment analysis of genes identified by at least two methods.

To uncover the genetic basis of prolificacy, we conducted selective sweep analysis between polytocous and monotocous DHS using four methods. Distinct selection signals were identified on multiple chromosomes, with $F_{ST}$, $\theta_{\pi }$ ratio, XP-CLR, and XP-EHH (Figure 3A) collectively highlighting key loci potentially associated with reproductive adaptation. In the top 1% of selected genomic regions, $F_{ST}$, $\theta_{\pi }$ ratio, XP-CLR, and XP-EHH, respectively, identified 470, 707, 1323, and 532 candidate genes (Figure 3B; Supplementary Table S5). *LOC101109111* and *LOC101109377* were consistently identified by all four methods, indicating their potential key roles in regulating litter size. Additional genes, such as *MUC1*, *MSH3*, *FNBP1L*, *TRIM46*, *CADM2*, and *CEP128*, were identified by at least three methods, suggesting they may be under multiple selection pressures and contribute to the genetic regulation of prolificacy. GO and KEGG analyses (Figure 3C; Supplementary Table S6) of candidate genes from selective sweep regions revealed enrichment in reproductive-related biological processes and pathways, including follicle development, hormonal regulation, ovarian steroidogenesis, insulin secretion, and circadian rhythm, suggesting their roles in regulating prolificacy and reproductive cycles.

### 3.5. Genome-Wide Association Analysis of Litter Size in DHS

**Figure 4:**
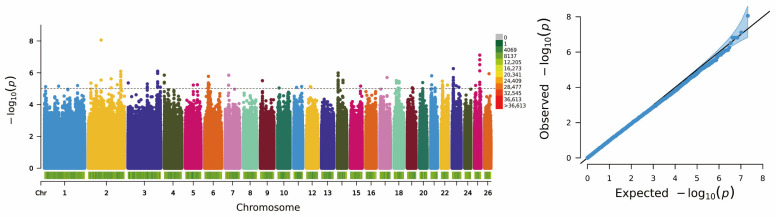
Manhattan Plot and QQ Plot of Genome-Wide Association Analysis for the prolificacy trait (polytocous vs. monotocous) of Dorper × Hu Hybrid Sheep. The phenotype represents the overall prolificacy status derived from the ewes’ reproductive records across three consecutive parities. The blue shadow in QQ Plot indicates the confidence interval.

A GWAS was performed using the GLM to identify genetic variants associated with litter size in DHS. The results are presented in Figure 4 (Manhattan and Q–Q plots). The Q–Q plot showed that all quantiles were distributed along the expected diagonal line (slope = 1), indicating that the population structure and genetic background were well controlled and the association signals were reliable. Using a threshold *p* < 10^−5^, the analysis yielded 140 significant SNPs, which annotated 83 candidate genes highly associated with litter size (Supplementary Tables S7 and S8). Among these, 12 SNPs met a more stringent significance level of *p* < 10^−6^. These 12 highly significant SNPs were mainly clustered on Chromosome 2 (2 loci) and Chromosome 25 (6 loci).

To validate the GWAS findings and identify potential functional mutations, targeted genotyping was performed on 200 DHS exhibiting three consecutive lambing records. The 10 candidate SNPs selected for MassARRAY^®^ genotyping were chosen based on two primary criteria: (1) their highly significant GWAS *p*-values, which ranged from 8.77 × 10^−9^ to 2.94 × 10^−6^, and (2) the known biological functions of their corresponding genes in reproduction and development. Specifically, genes harboring these selected SNPs included: *SLC24A2* and *MLLT3* (involved in cell signaling and gene transcription regulation); *PAX3* and *SGPP2* (related to embryonic development and lipid metabolism); *ABCA1* (affects steroid hormone biosynthesis and ovarian function); and *NRG3* and *KCNU1* (linked to neuroendocrine and sperm maturation processes). These 10 SNPs, located on Chromosomes 2, 3, 14, 23, 25, and 26, were then genotyped using the MassARRAY^®^ platform, which showed high detection rates ranging from 81.5% to 100% (Table 1).

Subsequently, using the full dataset of 200 DHS ewes, the GLM was used to analyze the association between each SNP genotype and the number of offspring (Table 2). The results revealed that two sites, g.88680390 C>A and g.18197516 T>C, showed significant associations with litter size (*p* < 0.05). Specifically, ewes carrying the AA genotype at g.88680390 exhibited consistently higher average litter sizes across the first to third parities compared with other genotypes. Similarly, the TT genotype at g.18197516 was associated with larger litter sizes than the heterozygous or homozygous mutant types. These results suggest that the identified loci may have favorable allelic effects on reproductive performance in DHS and could serve as molecular markers for the selection of high-prolificacy individuals.

## 4. Discussion

This study attempted to identify the genetic characteristics of DHS using whole-genome data, focusing on population genetic structure and genetic diversity. The results highlight the potential and value of DHS as a composite breed with distinct genetic features for future breeding and improvement programs.

Population genetic structure analyses supported the classification of DHS as a distinct genetic cluster. These findings indicate that DHS has developed a stable genetic foundation through hybridization and has diverged from its parental populations, rather than remaining an intermediate type. These findings align with previous studies on the Purunã composite cattle breed [29] and prolific Suffolk sheep [30], further demonstrating that systematic hybrid breeding can generate novel breeds with independent genetic structures through population recombination. This provides valuable insights for meat sheep-breeding strategies.

DHS displayed greater genetic polymorphism than both HUS and DPS. Genetic diversity indicators, including nucleotide diversity (*π*), *Ho*, *He*, *P_N_*, and MAF were all elevated in DHS, suggesting a greater accumulation of genetic variation during the hybridization process [31]. These findings imply that DHS harbors enhanced genetic potential for environmental adaptation and selective breeding. These observations are consistent with reports on high genetic diversity and low inbreeding in Luxi black-headed sheep [4], Bamei mutton sheep [5], and Creole goats [32]. This underscores the importance of hybrid breeding strategies in preserving genetic variability. ROH analysis showed that DHS predominantly contained short ROH segments, with relatively few long fragments. Combined with faster LD decay, this indicates a lower risk of inbreeding and a more relaxed population structure.

We identified overlapping candidate genes and enriched pathways in polytocous populations. *LOC101109111* and *LOC101109377* were consistently detected across all four selection methods. Although not extensively characterized previously, these genes may represent novel regulators of litter size. Several previously reported genes were also identified by three or more methods and appear to be under cumulative selection pressure. These include *MUC1*, which affects the uterine environment and embryo implantation and significantly influences litter size in pigs [33]; *PLCB4*, identified as a hub gene for litter size traits in sheep through meta-GWAS analysis [34]; *SIN3A*, which regulates chromatin remodeling during follicular development [35]; and *ELAVL2*, which is involved in oocyte maturation [36]. These genes are likely to play important roles in ovarian function and embryonic development. Moreover, the enrichment of pathways such as ovarian steroidogenesis [37], insulin secretion [38], Apelin signaling [39], and circadian entrainment [30] underscores the importance of coordinated endocrinological and developmental processes in prolificacy. These pathways have also been implicated in reproductive success across mammals, reinforcing the relevance of our findings.

In this study, we identified 140 significantly associated SNPs ($p<10^{-5}$) via GWAS and selected 10 highly significant candidate loci for validation. Candidate genes in the regions harboring these SNPs and their biological functions provide critical insights into the molecular regulatory mechanisms underlying the high fecundity of DHS. Among these, the most significant association signal ($p=8.77\times 10^{-9}$) maps to the g.88680390 C>A locus on chromosome 2, which is located in the intergenic region between the *SLC24A2* and *MLLT3* genes. Previous studies have shown that *SLC24A2* plays an important role in the reproduction of Cameroon’s native goats [40], suggesting a conserved function in sheep reproductive regulation. As a transcriptional activator, *MLLT3* can extend the transcription cycle of target genes and remodel chromatin during embryogenesis [41], and has been demonstrated to be involved in key processes of embryonic development [42]. Furthermore, significant differences in the expression levels of *MLLT3* were identified between the ovarian tissues of high- and low-fecundity Chongming white goats [43], further supporting its role in regulating reproductive traits. Another key candidate locus is g.18197516 T>C ($p=4.48\times 10^{-6}$), located within the *ABCA1* gene region. *ABCA1* plays a central role in transmembrane cholesterol transport, a precursor for steroid hormone synthesis. Studies have shown that *ABCA1*-deficient mice exhibit significantly reduced cholesterol ester levels in adrenal and ovarian tissues, leading to impaired reproductive capacity in homozygous female mice, characterized by fewer pregnancies and lower litter sizes [44]. Additionally, the g.36609350 G>C locus ($p=7.74\times 10^{-8}$) on chromosome 25 is associated with the *NRG3* gene, which has been identified as a candidate gene for litter size in sheep in multiple studies [44,45]. Although selective sweep analyses (e.g., $F_{ST}$, XP-CLR) and GWAS can both identify genomic regions associated with litter size variation, they capture distinct aspects of the underlying genetic architecture, resulting in incomplete overlap of detected signals. In this study, some loci (e.g., g.88680390 C>A) were identified by both methods—showing significant associations in GWAS while residing in regions with strong $F_{ST}$ and XP-CLR signatures—whereas some GWAS-significant SNPs were outside major selective sweep regions. This discrepancy stems from the fact that selective sweep analyses focus on identifying genomic regions shaped by historical or recent directional selection within populations [46], while GWAS are more sensitive to SNPs with measurable additive genetic effects on traits [47].

To validate the GWAS findings, we performed targeted genotyping on a larger population. We confirmed that two key SNPs—g.88680390 C>A (located in *SLC24A2/MLLT3*) and g.18197516 T>C (located in *ABCA1*)—are significantly associated with increased litter size. Specifically, ewes carrying the AA genotype at g.88680390 and the TT genotype at g.18197516 exhibited higher litter sizes. Consequently, we recommend prioritizing these SNPs to enhance reproductive rates. While this study provides valuable genomic insights, it has several limitations. Although the validation utilized 200 DHS individuals, this sample size remains limited for litter size. To improve confidence in these SNPs and facilitate reliable marker-assisted selection, future work should involve genotyping in larger DHS populations and integrating functional analyses (e.g., expression Quantitative Trait Loci or eQTL analysis) to elucidate the precise causal mechanisms of these markers.

## 5. Conclusions

In summary, this study systematically characterized the genetic structure and genetic diversity of DHS, providing comprehensive insights for its genetic breeding research. The results confirm that DHS has formed an independent and stable genetic background through systematic hybridization, with a distinct population structure and higher genetic diversity. Additionally, this study investigated selection signatures associated with litter size in DHS, identifying multiple candidate genes and pathways that may be involved in regulating reproductive performance. Furthermore, two SNP loci—g.88680390 C>A (located in the genomic region of *SLC24A2/MLLT3*) and g.18197516 T>C (located in the genomic region of *ABCA1*)—showed a significant association with litter size, providing potential molecular markers for the improvement of sheep fecundity. These results offer important references for the development of genomic selection and breeding strategies related to sheep reproductive performance.

## Figures and Tables

**Table 1:** MassARRAY^®^ SNP Genotyping Site Information.

SNP	Chromosome	Position	Mutation	Gene	GWAS *p*-Value	Detection Rate
**g.88680390 C>A**	2	88680390	C/A	*SLC24A2*, *MLLT3*	8.77 × 10^−9^	98.00%
**g.224575852 G>A**	2	224575852	G/A	*PAX3*, *SGPP2*	8.11 × 10^−7^	92.50%
**g.18197516 T>C**	2	18197516	T/C	*ABCA1*	4.48 × 10^−6^	100.00%
**g.205268702 A>G**	3	205268702	A/G	*LOC105613002*	7.96 × 10^−7^	97.50%
**g.205266796 T>C**	3	205266796	T/C		8.55 × 10^−7^	81.50%
**g.6708960 G>A**	14	6708960	G/A	*LOC105609364*, *DYNLRB2*	1.03 × 10^−6^	81.50%
**g.40389551 C>G**	14	40389551	C/G	*ZNF536*	2.94 × 10^−6^	97.00%
**g.7693935 T>C**	23	7693935	T/C	*DOK6*	2.98 × 10^−6^	87.00%
**g.36609350 G>C**	25	36609350	G/C	*NRG3*	7.74 × 10^−8^	99.50%
**g.31340267 C>T**	26	31340267	C/T	*KCNU1*	1.15 × 10^−6^	99.00%

**Table 2:** Association Analysis Between SNP Mutation Sites and Litter Size in 200 DHS using Three Consecutive Lambing Records.

SNP	Genotype	Number	Detection Number (Rate)	First Parity		Second Parity		Third Parity		Overall Mean	
				Litter Size	*p*-Value	Litter Size	*p*-Value	Litter Size	*p*-Value	Litter Size	*p*-Value
g.88680390 C>A	AA	29	196 (98.00%)	1.79 ± 0.12 ^a^	0.15	2.34 ± 0.17 ^a^	0.86	2.59 ± 0.14 ^a^	0.05	2.42 ± 0.11 ^a^	0.02
	CC	75		1.72 ± 0.07 ^a^		2.25 ± 0.09 ^a^		2.85 ± 0.06 ^a^		2.45 ± 0.05 ^a^	
	CA	92		1.58 ± 0.06 ^a^		2.26 ± 0.08 ^a^		2.62 ± 0.07 ^a^		2.24 ± 0.05 ^b^	
g.224575852 G>A	AA	64	185 (92.50%)	1.72 ± 0.08 ^a^	0.39	2.25 ± 0.11 ^a^	0.93	2.73 ± 0.07 ^a^	0.99	2.42 ± 0.06 ^a^	0.16
	GG	121		1.64 ± 0.05 ^a^		2.24 ± 0.07 ^a^		2.74 ± 0.07 ^a^		2.32 ± 0.05 ^a^	
g.18197516 T>C	CC	27	200 (100.00%)	1.37 ± 0.09 ^b^	0.01	1.85 ± 0.13 ^b^	0.01	2.30 ± 0.17 ^b^	0.00	1.98 ± 0.09 ^b^	0.00
	TT	148		1.74 ± 0.05 ^a^		2.34 ± 0.07 ^a^		2.80 ± 0.05 ^a^		2.44 ± 0.04 ^a^	
	TC	25		1.56 ± 0.10 ^ab^		2.24 ± 0.13 ^ab^		2.64 ± 0.16 ^ab^		2.24 ± 0.09 ^ab^	
g.205268702 A>G	AA	46	195 (97.50%)	1.57 ± 0.08 ^a^	0.48	2.00 ± 0.11 ^b^	0.02	2.72 ± 0.10 ^a^	0.97	2.23 ± 0.07 ^a^	0.15
	AG	98		1.68 ± 0.07 ^a^		2.32 ± 0.08 ^ab^		2.71 ± 0.07 ^a^		2.38 ± 0.05 ^a^	
	GG	51		1.71 ± 0.09 ^a^		2.43 ± 0.12 ^a^		2.69 ± 0.09 ^a^		2.41 ± 0.07 ^a^	
g.205266796 T>C	CC	37	163 (81.50%)	1.57 ± 0.09 ^a^	0.37	1.97 ± 0.12 ^b^	0.03	2.70 ± 0.11 ^a^	0.65	2.23 ± 0.08 ^a^	0.08
	CT	79		1.72 ± 0.07 ^a^		2.29 ± 0.09 ^ab^		2.78 ± 0.08 ^a^		2.44 ± 0.06 ^a^	
	TT	47		1.74 ± 0.09 ^a^		2.43 ± 0.12 ^a^		2.68 ± 0.09 ^a^		2.42 ± 0.07 ^a^	
g.6708960 G>A	GG	69	188 (94.00%)	1.65 ± 0.07 ^a^	0.76	2.13 ± 0.10 ^a^	0.08	2.55 ± 0.08 ^b^	0.01	2.27 ± 0.06 ^b^	0.05
	GA	119		1.68 ± 0.06 ^a^		2.34 ± 0.07 ^a^		2.82 ± 0.06 ^a^		2.42 ± 0.04 ^a^	
g.40389551 C>G	CC	80	194 (97.00%)	1.58 ± 0.06 ^b^	0.00	2.35 ± 0.09 ^a^	0.38	2.66 ± 0.07 ^a^	0.72	2.37 ± 0.06 ^a^	0.71
	GG	35		2.00 ± 0.12 ^a^		2.29 ± 0.13 ^a^		2.74 ± 0.14 ^a^		2.40 ± 0.08 ^a^	
	GC	85		1.61 ± 0.07 ^b^		2.18 ± 0.09 ^a^		2.74 ± 0.07 ^a^		2.32 ± 0.05 ^a^	
g.7693935 T>C	CC	18	174 (87.00%)	1.72 ± 0.19 ^a^	0.66	2.17 ± 0.20 ^a^	0.74	2.50 ± 0.15 ^a^	0.02	2.23 ± 0.11 ^a^	0.41
	TT	62		1.73 ± 0.08 ^a^		2.31 ± 0.11 ^a^		2.63 ± 0.09 ^a^		2.40 ± 0.06 ^a^	
	TC	94		1.64 ± 0.06 ^a^		2.22 ± 0.08 ^a^		2.87 ± 0.07 ^a^		2.39 ± 0.05 ^a^	
g.36609350 G>C	CC	71	199 (99.50%)	1.62 ± 0.08 ^a^	0.54	2.34 ± 0.10 ^a^	0.61	2.59 ± 0.09 ^a^	0.16	2.32 ± 0.06 ^a^	0.69
	GG	35		1.63 ± 0.09 ^a^		2.23 ± 0.12 ^a^		2.77 ± 0.12 ^a^		2.36 ± 0.08 ^a^	
	GC	93		1.72 ± 0.06 ^a^		2.22 ± 0.08 ^a^		2.78 ± 0.06 ^a^		2.39 ± 0.05 ^a^	
g.31340267 C>T	CC	60	198 (99.00%)	1.63 ± 0.08 ^a^	0.51	2.27 ± 0.11 ^a^	0.76	2.73 ± 0.08 ^a^	0.95	2.39 ± 0.06 ^a^	0.72
	CT	113		1.65 ± 0.06 ^a^		2.29 ± 0.07 ^a^		2.70 ± 0.07 ^a^		2.35 ± 0.05 ^a^	
	TT	25		1.80 ± 0.12 ^a^		2.16 ± 0.18 ^a^		2.72 ± 0.12 ^a^		2.30 ± 0.09 ^a^	

Different letters (^a^ and ^b^) indicate a significant difference between groups (*p* < 0.05). The same letters indicate no significant differences between groups (*p* > 0.05).

## Data Availability

The SNP variants generated in this study through variant calling using GATK are available via NutCloud at the following link: https://www.jianguoyun.com/p/DbjQdZgQ_tC_DRj9pIYGIAA (accessed on 1 July 2025).

## Supplementary Materials

The following supporting information can be downloaded at: https://www.mdpi.com/article/10.3390/ani15233505/s1, Table S1: Summary of Whole-Genome Resequencing Data Quality Before and After Filtering; Table S2: Summary of Whole-Genome Read Alignment Statistics to the Reference Genome; Table S3: Statistics of sample average sequencing depth and coverage ratio; Table S4: SNP location or type statistics; Table S5: Candidate genes associated with litter size in Dorper × Hu hybrid sheep identified by multiple selection signature methods and their overlaps; Table S6: GO and KEGG Enrichment Analysis of Candidate Genes Under Selection with litter size; Table S7: Results of Candidate SNP Loci Screening in Genome-Wide Association Analysis of Dorper × Hu Hybrid Sheep; Table S8: Site Primer Information Table. Figure S1: Heatmap of SNPs Density Distribution on Chromosomes.
