# Supplementary figures and images for: Whole-Genome Sequencing of Dorper × Hu Hybrid Sheep for Screening Selection Signatures Associated with Litter Size
Source: Animals (Basel). 2025 Dec 4;15(23):3505. doi: 10.3390/ani15233505 (PMC12691249; doi:10.3390/ani15233505)

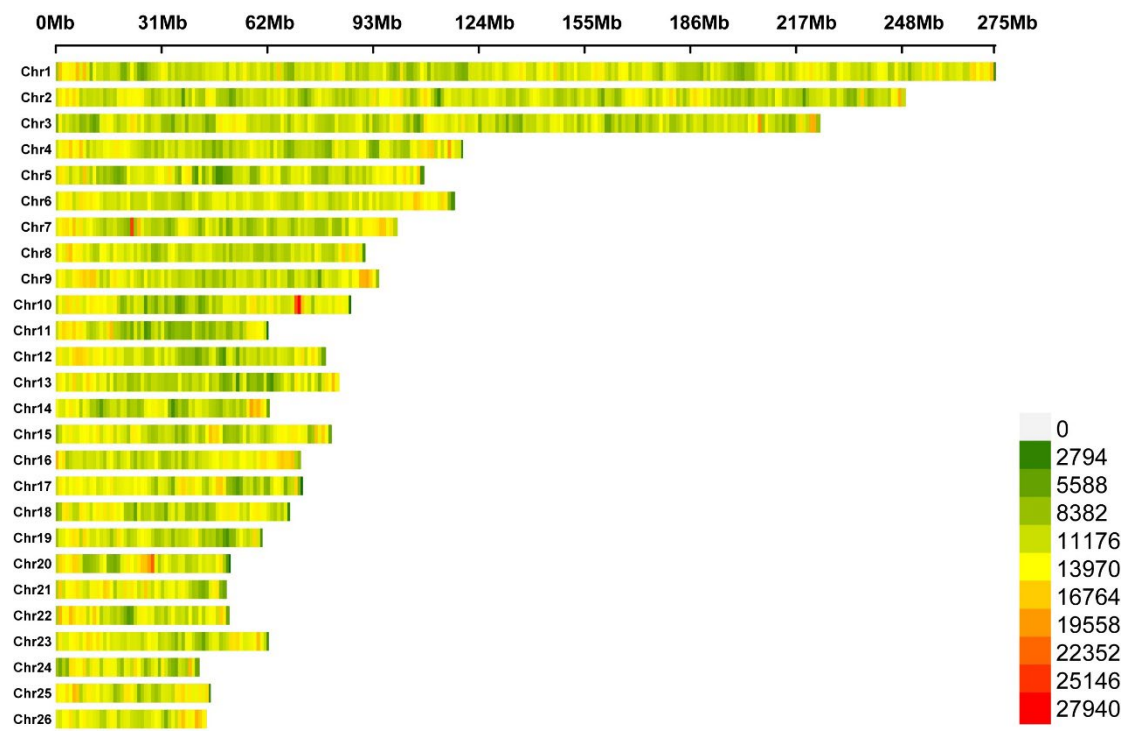

**Figure S1.** Heatmap of SNPs Density Distribution on Chromosomes

Supplement: Supplementary file 1 [file animals-15-03505-s001.zip › animals-3959142-supplementary/Supplement Figures.pdf]
